# P53 Mutations in Advanced Cancers: Clinical Characteristics, Outcomes, and Correlation between Progression-Free Survival and Bevacizumab-Containing Therapy

**DOI:** 10.18632/oncotarget.974

**Published:** 2013-05-01

**Authors:** Rabih Said, David S. Hong, Carla L. Warneke, J. Jack Lee, Jennifer J. Wheler, Filip Janku, Aung Naing, Gerald S. Falchook, Siqing Fu, Sarina Piha-Paul, Apostolia M. Tsimberidou, Razelle Kurzrock

**Affiliations:** ^1^ Department of Investigational Cancer Therapeutics (Phase I Clinical Trials Program), The University of Texas MD Anderson Cancer Center, Houston, TX; ^2^ Department of Biostatistics, The University of Texas MD Anderson Cancer Center, Houston, TX; ^3^ UCSD Moores Cancer Center

**Keywords:** P53 mutations, PTEN loss, bevacizumab, Li-Fraumeni syndrome

## Abstract

**Background:**

Mutations in the *p53* gene are amongst the most frequent aberrations seen in human cancer. Our objective was to characterize the clinical characteristics associated with *p53* mutation in patients with advanced cancer.

**Methods:**

We retrospectively reviewed and analyzed the clinical features and response to standard systemic therapy of 145 patients with documented tumor *p53* mutational status (mutant-type [*mtp53*] vs. wild-type [*wtp53*]) referred to the Clinical Center for Targeted Therapy.

**Results:**

Sixty-six (45.5%) patients had mtp53. Mutations in *p53* occurred more frequently in older patients (p= 0.015) and in Caucasians (p=0.024). The incidence of liver metastases was 69.2% vs. 43%, p=0.002 in *mtp53* and *wtp53*, respectively. PTEN loss by immunohistochemistry was found more frequently in *mtp53*-bearing tumors compared to *wtp53* (33.3% vs. 10%, p=0.007). The best progression-free survival (PFS) on standard systemic therapy was significantly longer with bevacizumab-containing regimens as compared to non-bevacizumab containing regimen in patients with *mtp53* (median 11.0 [95% CI 5.9-16.0], n=22 vs. 4.0 months [95% CI 3.6-5.7], n=35, p<0.0001) but not those with *wtp53* (median 5.0 [95% CI 2.0-7.6] vs. 6.0 [95% CI 4.0-7.5] months, p=0.318. The median overall survival from diagnosis in patients with *mtp53* and *wtp53* was 7.4 [95% CI 6.3-9.8] vs. 11.8 [95% CI 2.9-21.5] years, respectively (p=0.365).

**Conclusion:**

Patients with *mtp53* tumors were older at diagnosis, had more incidence of liver metastasis, and more frequent PTEN loss. The best PFS on standard systemic therapy was significantly longer with bevacizumab-containing regimens in patients with mutant *p53* tumors but not in those with *wtp53*.

## INTRODUCTION

Mutated *p53* is one of the most common genetic abnormalities detected in human cancers (1). The first alteration of the *p53* gene in human cancer was reported in 1989 (2). Since then, the *p53* gene has been a major research target in cancer biology and drug development, leading to the discovery of more than 30,000 reported mutations so far (3). Most *p53* mutations have been reported within centrally located hot spots in the DNA-binding domain (4).

As a major tumor suppressor gene, *p53* regulates the cell cycle, controls DNA repair mechanisms and activates apoptotic pathways (5). In addition, *p53* protein plays a role in regulating angiogenesis at least in part through direct binding to the hypoxia-induced factor-α (HIF-α) subunit, leading to HIF-α destruction (6). Under physiologic conditions, intracellular levels of *p53* protein are maintained at low levels by a complex network of proteins that include murine double minute 2 (Mdm2) (7). Targeting aberrant p53 has proven challenging. Mdm2 inhibitor molecules have demonstrated preclinical promise in a wide variety of tumors with wild-type p53 (8-10). Importantly, MK-1775, a potent and selective small molecule inhibitor of Wee-1 kinase (a tyrosine kinase that phosphorylates and inactivates CDC2 and is involved in G2 checkpoint signaling) selectively sensitizes tumors to DNA damaging agents, probably because *p53* is a key regulator in the G1 checkpoint and *p53*-deficient tumors rely only on the G2 checkpoint after DNA damage (11). It is currently being studied in phase II trials of ovarian cancer with *mtp53* (NCT01164995 and NCT01357161) (http://Clinicaltrials.gov). Overall, however, there is a paucity of molecules targeting *p53* mutations, and, because these mutations are found in over 50% of cancer, identifying ways to counteract them is important.

The clinical correlates of *p53* mutations malignancies have not been fully delineated. Preclinical data have shown that *p53* mutation accelerates cancer progression and increases tumor invasiveness and metastasis, which is not, however, the entire picture (12, 13). Here, we studied *p53* mutational status in patients with advanced malignancies referred to the Phase I Clinical Trials Program in The University of Texas MD Anderson Cancer Center. Our objectives were to identify clinical, prognostic and predictive characteristics associated with *p53* mutational status in advanced solid tumors.

## RESULTS

### Patient characteristics

The clinical and demographic characteristics of our patient population are summarized in Table 1. Overall, starting in May 2010, the *p53* mutational status of tumors was identified in 145 patients. The median age of the patients at diagnosis was 53.3 years (range, 14.5 to 75.3 years). Women comprised 50.3% (n=73) of the study population. Most patients were Caucasian (n = 115, 79.3%) and the remaining 30 patients were African American (n=15, 10.3%), non-white Hispanic (n= 11, 7.6%), or Asian (n= 4, 2.8%). The type of primary cancer varied among patients. The most common histologic subtypes were colorectal carcinoma (n=31, 21.4%), sarcoma (n=23, 15.9%), ovarian carcinoma (n=13, 9.0%) and melanoma (n=13, 9.0%). The histologic subtypes of patients reflected the pattern of referrals to the Phase I Clinic.

**Table 1:** Clinical characteristic of patients with p53-mutant and p53-wild-type disease (univariate analysis)

Characteristics	Total Patients, N=145	mtp53, N=66 (%)	wtp53, N=79 (%)	^*^ P-value
Age at diagnosis (median, range)	53.4 (14.5-75.3)	56.1 (22.2-72.6)	51.0 (14.5-75.3)	0.015
Age at diagnosis ≥60 years	45	29 (43.9%)	16 (20.3%)	0.004
Gender Male Female	72 73	34 (47.2) 32 (43.8)	38 (52.8) 41 (56.2)	0.740
Race White Non-White	115 30	58 (50.4) 8 (26.7)	57 (49.6) 22 (73.3)	0.024
Type of cancer Breast Colorectal Endometrial Head and Neck Lung Melanoma Ovarian Pancreatic Renal Sarcoma Other GI Other GU Others	3 31 5 12 8 13 13 3 3 23 9 9 13	1 (33.3) 20 (64.5) 3 (60.0) 5 (41.7) 6 (75.0) 3 (23.1) 7 (53.9) 3 (100.0) 0 (0.0) 8 (34.8) 5 (55.6) 3 (33.3) 2 (15.4)	2 (66.7) 11 (35.5) 2 (40.0) 7 (58.3) 2 (25.0) 10 (76.9) 6 (46.1) 0 (0.00) 3 (100.0) 15 (65.2) 4 (44.4) 6 (66.7) 11 (84.6)	
Site of metastasis^**^ Brain Liver Lung Retroperitoneum Bone Lymph node Soft tissue Peritoneal Adrenal Ovary Skin	17 79 103 34 51 110 30 48 15 19 16	8 (12.1) 45 (69.2) 49(75.4) 20 (30.8) 22 (33.9) 51 (78.5) 10 (15.4) 24 (36.9) 7 (10.8) 9 (28.1) 4 (6.2)	9 (11.4) 34 (43.0) 54(68.4) 14(17.7) 29 (36.7) 59 (74.7) 20 (25.3) 24 (30.4) 8 (10.1) 10 (25.0) 12 (15.2)	1.000 0.002 0.458 0.078 0.730 0.694 0.156 0.478 1.000 0.794 0.112
Median time from diagnosis to metastases (months)	8.9 (0 – 361.7)	7.6 (0 – 361.7)	9.9 (0 – 235.2)	0.536
Time from diagnosis to metastases >2 years	32 (22)	15 (22.7)	17 (21.5)	1.000
Median number of phase 1 therapies (range)	1 (0 – 4)	1 (0 – 4)	1 (0 – 4)	1.000
Median number of prior cancer therapies	3 (0 – 12)	3.5 (0 – 10)	3 ( (0 – 12)	0.080
Median number of metastases (range)^**^	3 (0 – 9)	4 (0 – 9)	3 ( 0 – 8)	0.080

* P-values are from Fisher's exact test or Kruskal-Wallis test, as appropriate.

** Patients may have multiple sites of metastasis and some patients may have unavailable data on their sites of metastasis.

The P-value in each row is computed for testing the association of each metastasis site and p53 mutation.

The median time from diagnosis to metastasis/recurrence was 8.9 months (range 0 – 361.7, months). Forty-eight (33.1%) patients had metastatic disease at diagnosis. The most common metastatic sites were the lymphatic system (n=110, 76.4%), lungs (n=103, 71.5%) and liver (n=79, 54.9%). The number of metastatic sites, as reported by the last available imaging studies, ranged from 0 to 9 (median 3). The total number of prior standard systemic therapies before referral to the Phase I Clinical Trials Program ranged from 0 to 9 (median 3) and the total number of phase I clinical trials that patients enrolled on ranged from 0 to 4 (median 1).

### P53 mutational status and clinical features

Of 145 patients, 66 (45.5%) had tumors harboring the *p53* mutation. The presence of a *p53* mutation was significantly associated with patient age at diagnosis. Cancer diagnosed at an older age was associated with a greater number of *p53* mutations than was cancer diagnosed at a younger age. At diagnosis, the median age of patients with *p53* mutated tumors was 56.1 years (22.2 -72.6 years) versus 51.0 years (14.5 - 75.3 years) for patients with *wtp53* tumors (p =0.0145). In addition, each year's increase in age at diagnosis was associated with a 4% increase in the odds of having a *p53* mutation (OR = 1.04, 95% CI 1.01 to 1.07, p =0.019).

The percentage of *p53* mutations varied with tumor type, as expected (Table 1). Of note, all three patients with pancreatic cancer were found to have *mtp53* tumors, and all three patients with renal cell carcinoma were found to have *wtp53*. Mutant p53 tumors metastasized more frequently to the liver than did *wtp53* bearing tumors (69.2% vs. 43.0%) (p = 0.002). In addition, *mtp53* tumors trended toward retroperitoneal metastasis compared to *wtp53* tumors (30.8 % vs. 17.7 %, p=0.078). There was no statistically significant difference in the percentage of metastases between *mtp53* and *wtp53* tumors to the lung, brain, lymph nodes or to any other metastatic sites.

Univariate analysis showed that *mtp53* tumors occurred more frequently in Caucasian patients (50.4%) compared to non-Caucasian patients (26.7%; p=0.024). However, there was no correlation between *p53* mutational status and gender, number of metastatic sites and time from diagnosis to metastasis.

The family history of cancer in patients with *mtp53* tumors was reviewed in patients’ electronic medical records. Three of 66 (4.5%) patients with *mtp53* met the criteria for Li-Fraumeni-like syndrome (14, 15). Among those three patients, two had sarcoma (ages at diagnosis 27 and 44) with multiple family members (first, 2^nd^ and 3^rd^ degree) having breast cancer, brain tumors, lung cancer and other malignancies. One patient with ovarian cancer (negative BRCA1/2 mutation) diagnosed at age 47 had multiple family members with breast cancer at a young age (42 and 48 years old), leukemia (20 years old), brain tumor and other malignancies.

### Types of p53 mutations and the co-existing molecular aberrations

Five (7.6%) patients were found to have two molecular abnormalities of the *p53* gene in two different exons and two (3.0%) patients were found to have two molecular abnormalities of the *p53* gene in the same exons. Insertion or deletion (indels) was seen in 7 (10.9%) patients. The most common *p53* mutations were seen in exon 5 (n=23, 33.3%), followed by exon 6 (n=14, 20.3%), exon 7 (n=12, 17.4%), exon 4 (n=9, 13.0%), exon 8 (n=9, 13.0%) and exon 9 (n=2, 3.0%).

The number of other molecular aberrations tested ranged from 0 to 3 (median 1). Insufficient tissue availability for testing was the main reason for the inability to test for other aberrations. However, some data on *PI3KCA*, *KRAS*, *BRAF*, *c-KIT* and *EGFR* mutations, and PTEN status assessed by IHC, was available for more than 50% of patients’ specimens. The number and proportions of all molecular aberrations and their relationships to *p53* mutational status are summarized in Table 2. PTEN loss by IHC, but not the other aberrations, was statistically correlated with *mtp53* (33%, 17/51 *mtp53* vs. 10%, 5/50 *wtp53* had concomitant PTEN loss, p= .007).

**Table 2:** p53 mutational status and co-existing molecular aberrations

	mtp53, N=66		wtp53, N=79		^*^ P-value
Molecular Aberrations	No. of Patients Tested	No. of Patients with aberration(%)	No. of patients Tested	No. of Patients with aberration(%)	
PTEN loss	51	17 (33.3)	50	5 (10.0)	0.007
BRAF mutation	44	1 (2.3)	56	3 (5.4)	0.629
PIK3CA mutation	56	3 (5.4)	66	9 (13.6)	0.221
KRAS mutation	48	7 (14.6)	56	9 (16.1)	1.000
EGFR mutation	40	1 (2.5)	46	0	0.465
c-KIT mutation	36	0	46	0	NA
PTEN mutation	19	3 (15.8)	23	0	0.084
NRAS mutation	29	4 (13.8)	33	1 (3.0)	0.176
GNAQ mutation	20	0	25	2 (8.0)	0.495
MET mutation	23	1 (4.4)	25	0	0.479

* P-values are from Fisher's exact test

### P53 mutational status and the longest PFS on conventional systemic treatment

We analyzed the longest PFS achieved on conventional systemic treatment (before referral for phase I treatment) for metastatic/recurrent disease according to *p53* mutational status and types of treatment regimens. Data on the longest times of PFS were available for 112 (77.2%) patients (57 patients with *mtp53* tumors and 55 patients with *wtp53* tumors). The median duration of the longest PFS for all patients was 5.9 months (range 0.6-49.9 months). The anti-angiogenic agent bevacizumab was included in the systemic treatment regimen in 25.9% (n= 29 out of 112) of patients with available data on the longest PFS. The inclusion of bevacizumab in the treatment regimen that had the longest PFS was significantly associated with *p53* mutation status (38.6% (n=22 out of 57) of those in the mutation group versus 12.7% (n=7 out of 55) of those in the wild-type group, p=0.0023).

**Figure 1a and b:**
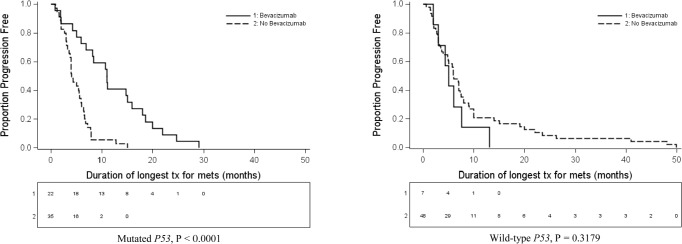
Kaplan Meier curve showing PFS on best standard systemic treatment in patients with *mtp53* comparing bevacizumab (n = 22) vs. non-bevacizumab containing regimens (n = 35) Kaplan Meier curve showing PFS on best standard systemic treatment in *wtp53* comparing bevacizumab (n = 7) vs. non-bevacizumab containing regimens (n = 48).

Among patients with *mtp53* tumors, the longest median PFS was 11.0 months (range 0.9 – 29.0 months) when the treatment regimen included bevacizumab (n=22) and 4.0 months (range 0.9–15.0 months) when the treatment did not include bevacizumab (n=35) (p< 0.0001) (Figure 1a). On the other hand, among patients with *wtp53*, there was no significant difference in PFS between bevacizumab-containing regimens (n=7) and regimens without bevacizumab (n=48) with a median of longest PFS being 5.0 (range 2.0-13.0) months vs. 6.0 (range 0.6-49.9) months respectively, p= 0.32) (Figure 1b).

In multivariate analysis, in patients with *mtp53*, significant predictors of a longer PFS included younger age (p=0.0096) and bevacizumab-containing regimen (p< 0.0001). In the *wtp53* group, none of the independent variables considered was predictive of a longer PFS (Table 3). Variables included in the multivariate model were patient age, race, and *p53* mutation status, whether the FDA recommended bevacizumb for the patient's tumor histology and whether the longest PFS was on a bevacizumab-containing regimen. In addition, we included the interaction term for *p53* mutation status and whether the patient was on a Bevacizumab containing regiment during longest PFS. We found that the interaction was statistically significant at p =0.0005 (Table 4).

**Table 3:** Multivariate Cox proportional hazards regression models predicting duration of longest PFS

Clinical Feature	mtp53			wtp53		
	Hazard Ratio	95% CI	P-value	Hazard Ratio	95% CI	P-value
Age^*^	0.96	0.94-0.99	0.010	0.99	0.97-1.02	0.651
Race (Non-Caucasian vs. Caucasian)	0.74	0.34-1.62	0.456	1.54	0.84-2.82	0.167
Tumor histology group§(Recommended vs. not recommended)	0.56	0.29-1.09	0.086	0.84	0.38-1.84	0.657
Bevacizumab-containing regimen (Yes vs. no)	0.21	0.09-0.454	<0.0001	1.82	0.661-5.02	0.247

* Age as continuous variable, § Recommended versus not recommended means dichotomized into tumor histologies for which bevacizumab is or is not FDA-approved

**Table 4:** Multivariate Cox proportional hazards regression model predicting duration of longest PFS

Clinical Feature	Hazard Ratio	95% CI	P-value
Age^*^	0.99	0.97-1.01	0.179
Race (Non-Caucasian vs. Caucasian)	1.32	0.83-2.09	0.243
Tumor histology group§ (Recommended vs. not recommended)	0.72	0.43-1.21	0.215
Bevacizumab -containing regimen (Yes vs. no)	2.50	1.00-6.29	0.051
P53 Status (Mutation vs. wild type)	2.65	1.57-4.47	<0.001
P53 mutation status and bevacizumab therapy interaction	0.15	0.05-0.44	<0.001

* Age as continuous variable, § Recommended versus not recommended means dichotomized into tumor histologies for which bevacizumab is or is not FDA-approved

**Figure 2:**
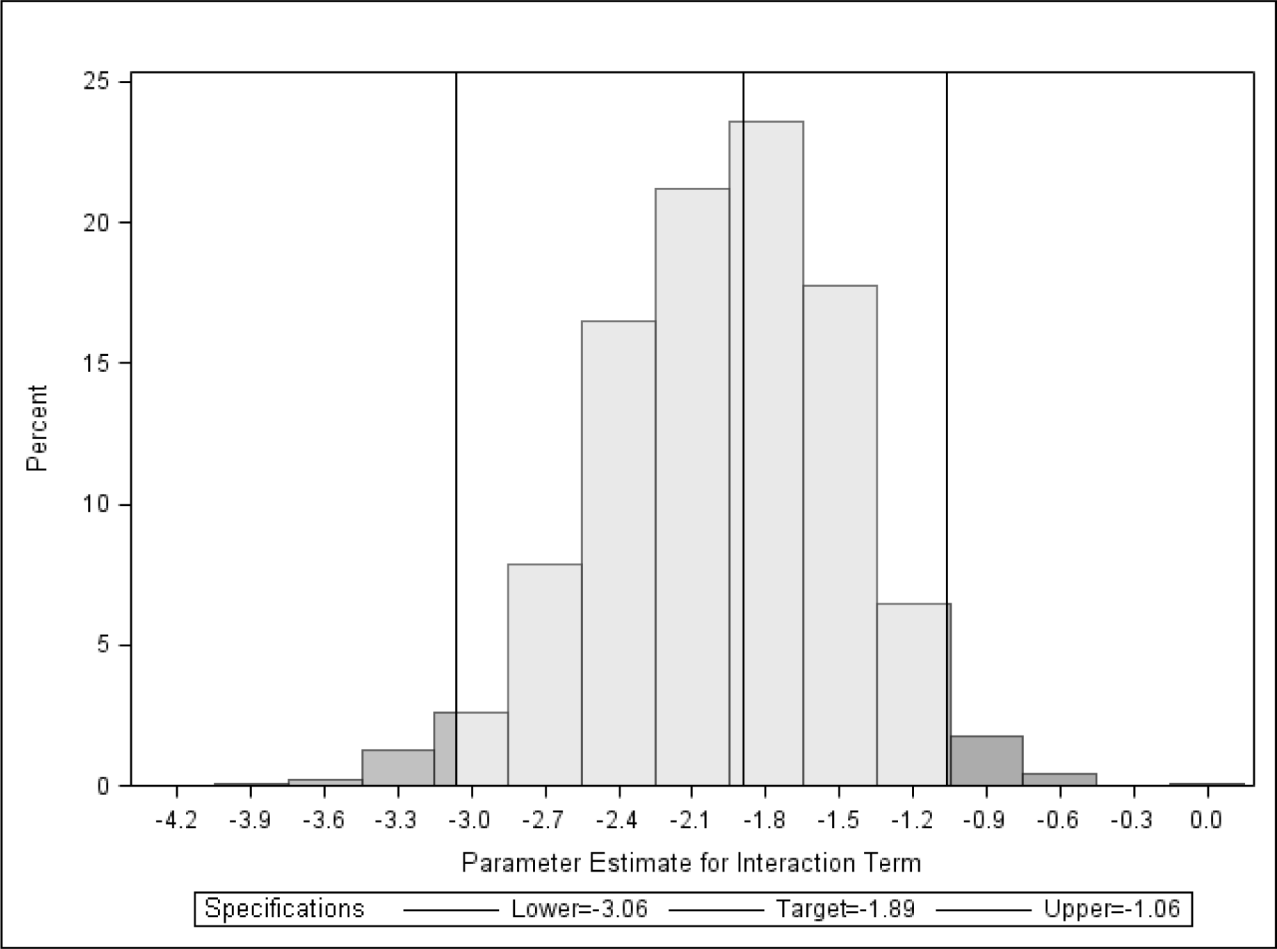
Distribution of the parameter estimate (-1.89, 95% CI -3.06, -1.06) for the interaction between P53 and bevacizumab using 1,000 bootstrap resamples with replacement

The bootstrap analysis and permutation test confirmed the independent prognostic value of interaction between *p53* mutational status and bevacizumab-containing treatment in predicting PFS in this data set (Figure 2). The 95% bootstrap confidence interval for the coefficient of the interaction between p53 mutation and the bevacizumab-containing treatment is (-3.02, -1.09). The P-value for testing zero interaction was 0.001 based on the permutation test.

Within the subgroup of patients who had colorectal cancer primary tumors, the longest PFS was available for 29 (n=20 with *mtp53* and n=9 with *wtp53*) patients. Among *mtp53*, the median longest PFS was 11.0 (95% CI: 7.0 -116.0) vs. 7.9 (95% CI: 3.3-15.0) months (p=0.16), if the patient received a bevacizumab-containing regimen or did not, respectively. Among *wtp53*, the median longest PFS was 5.5 (95% CI: 2.0-13.0) vs. 8.0 (95% CI: 4.9-22.0) months, by receipt of a bevacizumab-containing regimen or not, respectively (p=0.26).

### P53 mutational status and survival

Our patient population was heavily pretreated, with as many as 9 different systemic treatment regimens before referral to our Phase I Clinic and with as many as 5 different phase I clinical trials after referral. The difference between median overall survival from the time of diagnosis for patients with *mtp53* (n=66) and *wtp53* (n=79) was 4.4 years, but this difference was not statistically significant (7.4 (95% CI 6.3, 9.8 years) vs. 11.8 (95% CI 6.9, 21.5) years respectively, p =0.365). The median overall survival from phase I referral was 1.3 years (95% CI 0.9, 1.9 years) for patients with *mtp53* tumors and 2.6 years (95% CI 1.1, not attained) for *wtp5*3 patients, p=0.190.

## DISCUSSION

*P53* aberrations are frequent in cancer. The percentage of *p53* mutation varies by tumor type and ranges from 10% to 80% (http://p53.free.fr). Our patients had a 45.5% rate of *p53* mutation. We found that *mtp53* status was associated with more advanced age, results that are consistent with a previous study showing that *mtp53* occurred in older patients with rectal cancer (16). However, in contrast to our data, a previous study showed that *mtp53* breast cancer is three times more likely to occur in young women (age ≤40 years at diagnosis) as compared to wtp53 breast cancer (17).

Our analysis showed that *p53* mutations occurred more frequently in Caucasian patients in contrast to previously reported data which showed that *p53* mutation status did not differ between Caucasian and African-American patients (18, 19). However, the limited number of patients in our series precludes making definitive comparisons.

A role of *p53* mutation in cell migration and invasion (20) has been demonstrated in preclinical models (12, 13). These data could explain the higher percentage of patients with liver metastasis and the trend toward a higher percentage of patients with retroperitoneal metastasis seen in our *mtp53* tumors compared to those with wtp53 tumors. In fact, Tullo et al. previously showed that *mtp53* colorectal cancer had a greater number of metastatic liver lesions than *wtp53* colorectal cancer (21). Previously reported data showed that *p53* mutation is correlated with lymph node metastasis in pancreatic and prostate carcinoma (22, 23) which was not confirmed in our analyses.

Li-Fraumeni syndrome, characterized by heterozygous germline *p53* mutations and the occurrence of sarcoma, breast carcinoma, adrenocortical carcinoma, brain tumors and leukemia at a young age, is considered a very rare hereditary cancer syndrome and its characterization was initially based on a study of 12 families (24). The initial classical criteria to suspect Li-Fraumeni syndrome are very specific but have only moderate sensitivity (25). Since that seminal study, many other criteria have been uncovered, which increased the likelihood of detecting affected patients and families (25, 26). Gonzalez et al. showed that a germline mutation of *p53* resulted in a wider spectrum of tumors, as detected in 17% of the blood of cancer patients tested, and that 95% of patients in that study met the criteria for Li-Fraumeni or Li-Fraumeni-like syndrome (26). The family history of our patients with *mtp53* tumors showed that 3 of 66 patients (4.5%) met the criteria of Li-Fraumeni-like syndrome. Testing for germline mutations in this patient population might shed further light on this issue.

The role of the *TP53* gene in maintaining genomic integrity in mammalian cells has been previously described (27). Therefore, a greater number of molecular abnormalities would be expected to be associated with *mtp53*, which was difficult to confirm using our data due to the lack of available tissue for testing from a relatively large number of patients. Even so, our data showed a correlation between *mtp53* and *PTEN* loss by IHC. *PTEN* is an important inhibitor of the PI3K/Akt/mTOR pathway and loss of *PTEN* is associated with its activation (28). These findings are consistent with previously reported preclinical data showing coordinate regulation of the p53 and mTOR pathway (29). Stambolic et al. also showed that *PTEN* transcription is regulated by *p53* (30).

Of interest, our analysis demonstrated that the median longest PFS for patients with *mtp53* was 11 (95% CI 5.9, 16.0) months if bevacizumab was included in the treatment regimen versus 4 (95% CI 3.6. 5.7) months if it was not (p<.0001). These data complement previously published preclinical data demonstrating a correlation between *mtp53* and increased VEGF expression and vessel density in head and neck tumors (31), breast carcinoma (32, 33) and stromal cell of the bone marrow in leukemic patients (34). Other authors also reported the role of *p53* protein in inhibiting angiogenesis and neovascularization (35, 36). Additionally, the correlation between *p53* mutation and neovascularazation has been observed in various tumor cell lines and xenograft tumor models (6). On the other hand, Ince et al. showed that, in patients with colorectal cancer treated with bevacizumab, *p53* mutational status was not associated with survival (37). However, our multivariate analysis showed that within the group of patients with *mtp53*, treatment with a bevacizumab-containing regimen was an independent factor associated with longer PFS. Whether or not bevacizumab was associated with prolonged PFS in the *wtp53* group could not be accurately assessed because of the small number of patients. There are methodology differences between the study of Ince et al. and ours that may account for the appearance of discrepant results. The most important difference is that we focused on analyses of PFS while Ince et al. analyzed the overall survival. Hence, two studies cannot be compared.

Our analysis has several important limitations. First, diverse histologies were analyzed with small numbers of patients in each diagnostic category, precluding our ability to determine the relationship between *p53* and other factors within disease subgroups. On the other hand, our findings could imply that the impact of bevacizumab on PFS in *mtp53* tumors is not dependent on histology. Studies with larger numbers of patients are needed for confirmation. The retrospective nature of the study is also a limitation. There may also be a selection bias, because we analyzed only patients with metastatic disease and cannot ascertain the clinical behavior of tumors in patients whose disease never metastasized. The absence of randomization in regard to PFS also limits the firmness with which conclusions can be made, even in the presence of a multivariate analysis. Because of these limitations, this study is best viewed as exploratory and further studies are warranted to validate our findings.

In conclusion, *mtp53* tumors appear to have distinct biological associations, including older age at diagnosis, increased liver metastasis and association with PTEN loss as compared to *wtp53* tumors. When analyzed retrospectively, patients with *mtp53* had significantly longer PFS if bevacizumab was included in their therapeutic regimens than if it was not. Prospective studies are warranted to further investigate these observations.

## METHODS

### Patients

We investigated the *p53* mutation status of consecutive patients with advanced tumors with available tissue referred to the Clinical Center for Targeted Therapy in the Department of Investigational Cancer Therapeutics (Phase I Clinical Trials Program) at The University of Texas MD Anderson Cancer Center starting from May 2010 through March 2011. Out of 832 patients, 145 were tested for *p53* mutations. Other patients were not tested because of lack tissue availability. Patients referred to the Phase I Clinic were of various ages and histology types. Upon presenting to the Phase I Clinic, all patients had either metastatic or locally recurrent/advanced disease and were not candidates for treatment that was associated with a clinically significant improvement in survival. In general, no therapy that prolonged survival by more than three months was available. To identify the clinical impact of *p53* on cancer behavior and response to treatment, we compared the demographics, clinical characteristics, and responses of patients to their best systemic treatment before referral to the Phase I Clinic based on their *p53* mutational status. The study was performed in accordance with MD Anderson Institutional Review Board guidelines.

### Tissue samples and mutational analysis

Available tissue from diagnostic and therapeutic procedures was used to assess for molecular aberrations including *p53* mutational status. Tissue includes paraffin-embedded tissue blocks, formalin-fixed archived specimens, and/or fine needle biopsy aspiration. All pathology was centrally confirmed at MD Anderson. Testing for all molecular aberrations including *p53* mutations was performed in a Clinical Laboratory Improvement Amendment (CLIA)–certified Molecular Diagnostic Laboratory within the Division of Pathology and Laboratory Medicine at MD Anderson, and was done on any available sample with adequate tissue for testing.

DNA was extracted from microdissected tumor sections and analyzed using a polymerase chain reaction (PCR)-based DNA sequencing method for *p53* mutation within the most common area (exons 4-9) (38).

Whenever tissue was available, other mutations such as *EGFR* (exons 18 - 21)(39), *KIT* (exons 11, 13 and 17) (40), *PIK3CA* (exons 9 and 20) (41), *NRAS* and *KRAS* (exon 1 and 2) (42, 43), *BRAF* (exon 15) (42), *GNAQ* (44) and *MET* mutations were also tested (45). PTEN loss was assessed using immunohistochemistry (46) (monoclonal mouse anti-human PTEN, clone 6H2.1; Dako®, Denmark).

### Statistical analysis

Statistical analysis was carried out by our statisticians (CLW & JJL). Clinical variables were assessed by reviewing patient electronic medical records. The variables analyzed were age, gender, race, site and number of metastases, site of mutation, presence of other aberrations (*PI3KCA, NRAS, KRAS, EGFR, BRAF, PTEN, GNAQ* and c-*KIT* mutations and PTEN loss by immunohistochemistry [IHC]), the systemic therapy with the longest progression-free survival (PFS) before referral to the phase I clinic; and survival from date of diagnosis. The family history of each patient with mutated *p53* tumors was reviewed and analyzed to determine if patients met Li-Fraumeni or Li-Fraumeni-like syndrome criteria (25).

Patient demographics and clinical characteristics were summarized using simple descriptive statistics including frequencies, percents, and medians. Associations between *p53* mutation status and patient characteristics were assessed using Fisher's exact test and the Kruskal-Wallis test, as appropriate. Odds ratios and 95% confidence intervals were computed using univariate logistic regression models. PFS and overall survival were analyzed by the method of Kaplan and Meier. PFS while on systemic therapy before referral to the Phase I Clinic was the time interval between the start of therapy to the first observation of disease progression (as determined by clinical or radiological findings) or death whichever came first. For the overall survival, patients who were alive at the end of follow-up were censored at the date of their last follow-up. Survival probabilities were compared among subgroups of patients using the log-rank test. Univariate and multivariate Cox proportional hazards regression models were fit to assess the association between the time-to-event endpoints and patient characteristics, including *p53* mutation status. A subanalysis was conducted including only patients with colorectal cancer primaries. This subanalysis was performed because colorectal cancer was the largest histologic subgroup, and in addition, bevacizumab is approved for this indication. A post-hoc bootstrap procedure was used to verify the results obtained with the Cox proportional hazards model. From the original data set, we constructed 1000 replicate data sets using resampling with replacement. Thus, the size of the replicate data sets remained the same as in the original cohort, but the composition differed. The distribution of regression parameters in the bootstrap samples was calculated to form the 95% confidence interval. In addition, the P-value based on the permutation test was computed by comparing the model estimate to the estimates under the null hypothesis by shuffling the mutation status 100,000 times in the original data set to create the null distribution. P-values less than 0.05 were considered statistically significant. All statistical analyses were conducted using SAS software (v 9.2).
